# Adjuvant-free immunization with infective filarial larvae as lymphatic homing antigen carriers

**DOI:** 10.1038/s41598-020-57995-8

**Published:** 2020-01-23

**Authors:** Catherine Card, David S. Wilson, Sachiko Hirosue, Marcela Rincon-Restrepo, Alexandre de Titta, Esra Güç, Coralie Martin, Odile Bain, Melody A. Swartz, Witold W. Kilarski

**Affiliations:** 1 Institute of Bioengineering and Swiss Institute for Experimental Cancer Research (ISREC), École Polytechnique Fédérale de Lausanne (EPFL), Lausanne, Switzerland; 2 Institute for Molecular Engineering, University of Chicago, Chicago, IL USA; 3 UMR7245, MCAM, Museum National d’Histoire Naturelle, Paris, France

**Keywords:** Fluorescence imaging, Immunological techniques, Adjuvants

## Abstract

Controlled infection with intestinal nematodes has therapeutic potential for preventing the symptoms of allergic and autoimmune diseases. Here, we engineered larvae of the filarial nematode *Litomosoides sigmodontis* as a vaccine strategy to induce adaptive immunity against a foreign, crosslinked protein, chicken egg ovalbumin (OVA), in the absence of an external adjuvant. The acylation of filarial proteins with fluorescent probes or biotin was not immediately detrimental to larval movement and survival, which died 3 to 5 days later. At least some of the labeled and skin-inoculated filariae migrated through lymphatic vessels to draining lymph nodes. The immunization potential of OVA-biotin-filariae was compared to that of an OVA-bound nanoparticulate carrier co-delivered with a CpG adjuvant in a typical vaccination scheme. Production of IFNγ and TNFα by restimulated CD4+ cells but not CD8+ confirmed the specific ability of filariae to stimulate CD4^+^ T cells. This alternative method of immunization exploits the intrinsic adjuvancy of the attenuated nematode carrier and has the potential to shift the vaccination immune response towards cellular immunity.

## Introduction

Here, we show that it is possible to turn infectious L3 larvae of parasitic filaria nematode *Litomosoides sigmodontis* into a single carrier for both a natural filarial adjuvant and a non-filarial chicken protein (ovalbumin, OVA). OVA bound to filaria cuticle was carried out to the lymph node, where it induced OVA-specific immunity. Because all nematodes share the same mechanism of lymphatic entry any non-parasitic free-living nematode can be used in place of potentially hazardous filariae.

Most clinically used vaccines induce Th2-type humoral protection, and the titer of protective antibodies determines their efficacy^1^. However, combating metastatic tumors and chronic viral infections requires, among other things, the stimulation of Th1-type cellular immunity^2^. For example, attenuated viral and DNA vaccines that specifically stimulate Th1-type responses are currently in clinical trials^3^, while preclinical trials have shown that nanoparticle vaccines can have similar activity as viral and DNA vaccines in mice^4^. In consequence, there are few effective therapies against a large number of intracellular parasites or metastatic cells^5^. The limited repertoire of potential vaccination strategies prompted us to examine an unconventional approach, that is, to use live filaria larvae as a carrier of intrinsic adjuvant and antigen of choice.

Lymphatic filariasis is caused by parasitic agents that target the mammalian lymphatic system^6^. Filariae migrate through the pre-nodal afferent lymph and can dwell in the capsular and subcapsular sinuses of lymph nodes for up to 15 years; however, only 30% of infected individuals develop clinical disease^7–9^. One reason for the absence of clinical disease is helminth-induced immunosuppression, which hides parasites from detection by the immune system^10^. During chronic infection, parasites induce immunosuppression in the host by activating worm antigen-specific CD4^+^ CD25^+^ Foxp3^+^ regulatory T cells (Tregs), resulting in immunotolerance by Th2 lymphocyte anergy. In addition, the alternative activation of innate immunity occurs in the form of M2 macrophage polarization, which limits infection and tissue damage. Furthermore, a modified Th2 response leads to the production of non-nematode-specific polyclonal IgE and anti-inflammatory IgG4 in humans and IgG1 antibody isotypes in mice^11^. The immune system is consequently unable to kill adult lymphatic filariae, but it is also prevented from causing inflammatory damage to the lymphatic vessels^12^.

Counterintuitively, a large number of microfilariemic individuals with an asymptomatic filarial infection have markedly increased flow in the lymphatic system occupied by adult filariae, and lymph vessels remain patent as long as the nematodes are alive^7^. Live adult filariae induce the dilatation and wall hypertrophy of lymphatic channels and subcapsular sinusoids, but with nematode death and concomitant opportunistic and recurrent bacterial and fungal infections, lymphatic vessels become severely injured and occluded, leading to hydrocele and lymphedema^9,13^. Over time, connective tissue builds up, causing the characteristic symptoms of elephantiasis^12^. The eventual immune response against the nematode parasite is largely a consequence of the release of *Wolbachia*, a bacterial endosymbiont of filariae^14^. *Wolbachia* are invertebrate-specific intracellular bacteria that live in obligate mutualistic symbiosis with most filarial parasites, controlling their fertility and development^15^. When released by the necrotic cells of dying filariae, these gram-negative bacteria induce IgG2 and cellular Th1 and Th17 immune response against its own but also filarial antigens^8,16,17^.

Hence, *Wolbachia* can be considered a natural adjuvant for filaria-associated antigens that skews the immune response towards a Th1 phenotype^16^. In addition to the release of intracellular bacteria, unmethylated CpG sequences from immature filarial nematode DNA^18^ might stimulate immune cells via TLR9 and additionally promote Th1 immunity, as shown in mice^19^. Finally, the prolonged release of antigens from decomposing dead larvae might act as a particulate adjuvant and stimulate anti-nematode and anti-ovalbumin (OVA) Th2-type immunity^20^. Regardless of the specific adjuvant, the dying larvae induce a Th1-type immune response against *Wolbachia* and their own antigens^16^. However, aside from a single study showing the mitogenic effect of DNA from *Caenorhabditis elegans* on B cells^21^, the adjuvant properties of parasitic nematodes remain unexplored in the literature.

Here, we exploit the lymphatic homing properties^22^ of filariae to deploy a customized antigen payload into the core of the immune system. The labeling process transformed self-guided skin-to-lymph node infective larvae into vaccine carriers. The intrinsic adjuvancy of dying filariae facilitates the stimulation of OVA-specific immunity via the release of nonpathogenic intracellular *Wolbachia* bacteria^17,23^, methylated CpG sequences of discretely methylated nematode DNA^18^, or the slow-release of OVA from particulates of the protease-resistant cuticle^20,24^. Furthermore, cuticle collagens are resistant to proteolysis because of the high concentration of unique, nonreducible di- and tri-tyrosine bonds^24^. This protection from rapid clearance delays the decomposition of large, millimeter-sized larvae, providing support for prolonged immune stimulation via the gradual release of particulate antigens that can also act as an adjuvant^25^. Finally, the simultaneous release of antigen and intrinsic adjuvant directly in the lymph could farther amplify the immune responses^26,27^.

Importantly, pathological conditions linked to filariasis are induced only by large adult nematodes and microfilariae present in high numbers in the skin or blood during the patent (symptomatic) disease period^28^, with the first stage of the prepatent infection, the migratory period (e.g., before filarial L3 larvae reach their final habitat), being entirely asymptomatic^29–31^. Therefore, the prior attenuation of larvae alone should be sufficient to ensure the safety of a potential treatment, eliminating the need to choose a parasite with pre-existing host incompatibility (inability to develop a patent, reproductive infection)^32^.

## Results

### Intravital labeling of filariae has no immediate detrimental effect on larval behavior

**Figure 1:**
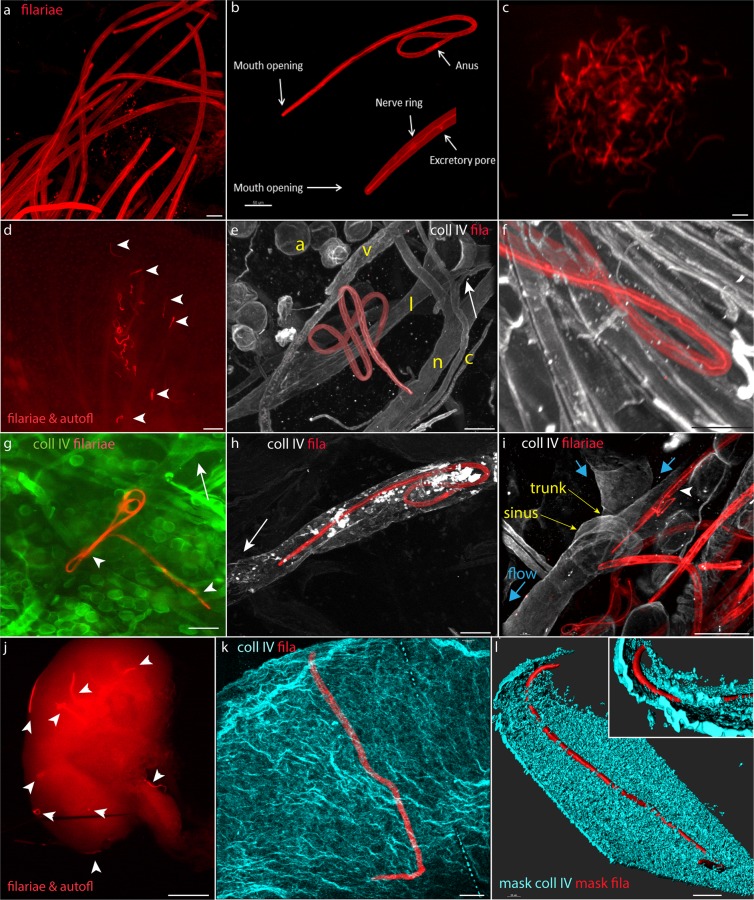
Chemical modification of filarial proteins has no adverse effect on filarial physiology. (**a)** Confocal Maximum Intensity Ptojection (MIP) of TRITC-labeled filariae. Labeled larvae were fluorescent and readily detectable by fluorescence microscopy. The labeling was uniform between live filariae. (**b)** MIP image of the fluorophore labeling of a larva revealed various anatomical organs of the nematode. The intestine begins with the mouth opening located at the tip of the larva and ends at the anus valve approximately 9/10 of the way along its length. The nerve ring surrounds the pharynx and is located in front of the asymmetric excretory pore at the anterior end of the larva. (**c**) A swarm of FITC-labeled and actively moving filaria (see also Video 1). For consistency, FITC-labeled filariae are shown in red. (**d**) Exposed ear dorsal dermis with the site of filarial injection. Approximately half of the injected filaria remained at the inoculation site 3 hours after injection. Arrowheads point to labeled larvae that spread within the skin. (**e**) MIP images of filaria migrating in the skin. The larva is wrapped around the lymphatic collecting vessel. Stationary structures in the skin revealed with basment membrane (BM) staining.: a, adipocytes; l, lymphatic collector; v, venule; c, capillary; n, nerve. The arrow points to the valve of the lymphatic precollector. (**f)** MIP image of filaria that left the dermis and entered the subcutaneous muscle (densely packed parallel BM structures surrounding individual muscle fibers). Subcutaneous lymphatics join the dermal lymphatic system before entering the parotid lymph node^35^. (**g)** Epifluorescence image of the ear dermis stained for collagen IV (green). Two filariae in the lymphatic collectors. Arrowheads point to larval heads. The arrow points to the lymphatic valve. (**h,i)** MIP images of filariae within lymphatic collectors. (**i)** Only the filaria marked with the arrowhead is located within the lymphatic collector. Collectors are distinguished from other stationary cutaneous structures by the presence of valves, sparse muscle coverage and the uneven diameter of the vessel along its length^13^. Blue arrows point to the direction of the lymph flow that was inferred from the morphology of the collector valves. (**j)** Epifluorescence image of the labeled filariae that reached the draining lymph node 1.5 hours after inoculation in the afferent dermis of the mouse ear. Strongly stained larvae stand out from the autofluorescence of the thick lymph node. Arrowheads point to individual larvae. (**k)** MIP image of the lymph node capsule and a single filarial larva. (**l)** M asking of the collagen IV (cyan) and filaria (red) signal with intensity surfaces revealed the subcapsular location of the larva. **Inset**. The larva is located between two layers of the basement membrane of the subcapsular sinus. A-B, E-F, H-I, and K-L - maximum projections of the fixed and cleared whole-mount tissue preparations imaged with a confocal microscope; C, D, G, and J - epifluorescence images of live surgically exposed (C) skin, or *ex vivo* images (D, G, and J). Scale bars A-B, E-I, K-L, 50 μm; C-D, G, and J, 500 μm.

First, we verified that the permanent, covalent modification of filarial proteins is not immediately toxic to *L. sigmodontis* larvae. The modification of amino groups in proteins represents the most straightforward chemistry that can be safely performed under physiological conditions of living tissue^33^. To assess the efficiency of the chemical cross-linking of amino groups in live filarial larvae, we labeled filariae with isothiocyanate derivatives of tetramethylrhodamine (TRITC) or fluorescein (FITC) fluorophores. The staining patterns of TRITC and FITC were equivalent, but to minimize the phototoxic effect of the filaria-bound fluorophore in ambient light, we routinely used TRITC^34^. The fluorophore labeling of filariae produced a strong signal that was uniform between filarial larvae and revealed various anatomical structures of the nematode (Fig. 1a). The intestine begins at the mouth opening located at the tip of the nematode and ends at the anus valve, approximately 9/10 the length of the body from the tip. In addition to surface proteins being labeled, a structure that is entirely hidden within the body, the nerve ring, was also stained with the amine-reactive fluorochromes (Fig. 1b). Despite the strong labeling, the chemical modification of the protein amino groups did not noticeably affect filarial behavior *in vitro*. Similar to the unlabeled larvae, the labeled filariae moved vigorously and after sedimentation, participated in a swarm (Fig. 1c; Video 1). FITC labeling was indirectly toxic to the filarial larvae, which died rapidly after exposure to blue light (Video 2). Therefore, all handling of the labeled larvae was performed in darkness, and when applicable, larvae were labeled with red dyes (Alexa Fluor 546- and Alexa Fluor 647-labeled streptavidin).

In mice, filariae overlaid with culture media on the surface of the exposed dermis could probe the exposed skin but were unable to enter the dermis (Video 3). When injected intradermally, the labeled filariae migrated within the dermis for at least 8 hours (Fig. 1e,f and Videos 4,5). A fraction of these larvae then entered the collecting lymphatics (Fig. 1g–i), identified by their sparse smooth muscle coverage and uneven diameter along the vessel length due to the presence of afferent and narrow trunks and post valve sinuses^34^ (Fig. 1i). As shown in Fig. 1j labeled L3 filariae, also reached a draining parotid lymph node, typically located approximately 3.5 cm away from the site of filarial injection^35^. Within the lymph node, larvae occupied space in the subcapsular sinus (Fig. 1k,l), which is the anatomical location typically favored by lymphatic filariae^7,9^. These results indicate that the chemical modification was not immediately toxic to the larvae and did not affect their physiological behavior, as filariae were still able to home to lymphatic vessels and migrate within them to the draining lymph node.

### Protein conjugation to live filariae

In addition to the potential toxicity of the chemical modification of surface proteins, we were concerned about the size of the attached molecules. Size alone can have a detrimental effect on physiological functions, for example, through the steric hindrance of large molecules that can disrupt receptor-ligand binding^36^. Therefore, we explored two different bioorthogonal cross-linking techniques, the S-4FB/S-HyNic and amine-reactive-biotin/streptavidin/amine-reactive-biotin systems, which both enable the *in vivo* formation of non-dissolvable bonds between proteins. This approach allowed us to couple antigens and filariae after the independent activation of the antigen and the nematode proteins. As with FITC or TRITC labeling, immobilizing OVA-streptavidin protein complexes on filariae did not affect their behavior, and the larvae remained active and alive for up to 5 days after *in vitro* staining (Videos 6,7). In the long term, chemical labeling was toxic to larvae, as all filariae died 3 to 5 days after staining. This mortality contrasted that of the unlabeled *L. sigmodontis* that survived in culture for two weeks. Hence, chemical labeling of filariae did not abolish the infective capability of larvae but did ensure that all nematode parasites would die within the skin or lymphatic system before establishing a patent infection, which happens 7 to 9 days after inoculation^37^.

**Figure 2:**
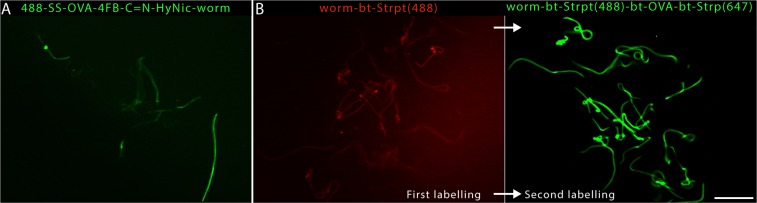
Bioorthogonal labeling of filariae with OVA protein bound only to the cuticle of larvae. Epifluorescence stereomicroscope images. **(A**) HyNic-4FB-OVA-Alexa 647 labeling produced weak and uneven filarial staining between larvae. (**B**) 2 Left The immobilization of Alexa-488-streptavidin on live filariae. After being biotinylated *in vivo*, larvae were incubated with Alexa-488-streptavidin. The lengthy exposure produced the high background intensity of the image. (**B)** Right. The same stained larvae were incubated with biotinylated OVA that was stained with Alexa 647-streptavidin. The second round of labeling produced an 11-fold stronger signal than the initial labeling of filarial nematodes (“worms”) with streptavidin-488 shown in (**B**) (left). In contrast to direct FITC or TRITC, streptavidin-OVA labels the nematode cuticle but not its internal anatomical structures. Scale bar, 400 µm.

Prefunctionalizing the larvae with aromatic hydrazines (S-HyNic) and OVA with a corresponding aromatic aldehyde (S-4FB) followed by subsequent Schiff base-mediated specific conjugation^38^ of the activated OVA and live larvae resulted in the weak and uneven labeling of filariae, with the larvae containing random high-intensity fluorescent patches on their bodies (Fig. 2A). Additionally, the pre-functionalization of filarial proteins with S-4FB was immediately toxic to the filaria, with 50% of larvae dying during the labeling process. Injured and dying larvae were more intensely labeled than live filariae (immotile larvae in Video 6), which was likely due to the loss of the integrity of the cell membrane and exposure of intracellular proteins in necrotic cells.

In contrast, the biotinylation of larval proteins with biotin EZ Link sulfo-N-hydroxysuccinimide ester (NHS- biotin) and subsequent crosslinking of biotinylated OVA to filariae by a tetrameric streptavidin linker did not noticeably harm the larvae and generally yielded uniform labeling of filariae with biotinylated OVA, as determined by the subsequent staining of the filarial larvae-bound OVA with the sequentially applied Streptavidin-Alexa Fluor 488 (Fig. 2B, left). Additionally, because of the tetrameric nature of streptavidin, the second layer of biotinylated OVA could be bound to the filaria-biotinylated OVA-streptavidin complex. This “sandwich” labeling amplified the deposition of total biotinylated OVA on the surface of the larva, as inferred from the 11-fold increase in signal intensity with the subsequent second staining of biotinylated OVA using differently labeled streptavidin (corrected for differences in camera sensitivity and exposure times for different wavelengths, Fig. 2B, right). On average, this labeling method immobilized 115 ng of OVA on the surface of a single nematode, as determined through the densitometric detection of biotinylated OVA by a Western blot of the filarial lysate.

In contrast to the single-step labeling of larvae with small molecular hydrophobic dyes (FITC and TRITC), sandwich labeling enabled proteins deposition only on the filarial cuticle, as internal structures of the larvae remained unstained. The dissimilarities in the staining pattern probably reflect the differences in size and hydrophobicity between proteins and the fluorescent dyes, which can diffuse freely through cell membranes^39^ and stain proteins inside live filariae.

Additionally, isolated from soil, non-parasitic nematodes of various sizes and trophic behavior could also survive intravital labeling^22^. When delivered tyo the skin, a fraction of free-living nematodes migrated through the dermis and enter lymphatic (Supplementary Fig. 1).

### OVA-conjugated filariae induce an antigen-specific immune response

**Figure 3:**
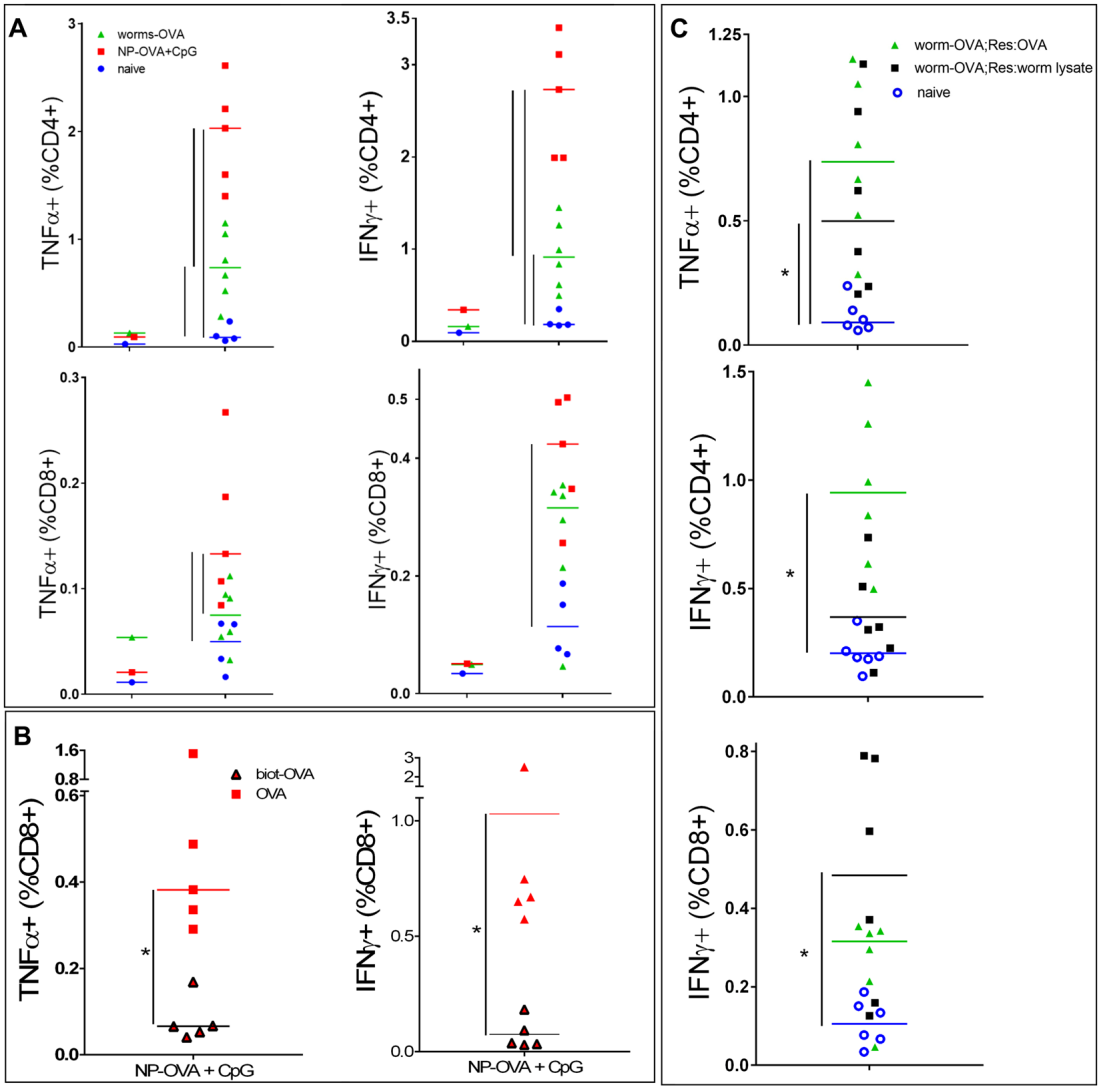
OVA-conjugated filariae induce OVA-specific T cell responses. Splenocytes were isolated from naive mice or mice vaccinated with either OVA-conjugated nanoparticles (NP-OVA + CpG) or OVA-conjugated filariae and were restimulated with OVA or filarial lysate for 6 hours. (**A)** Restimulation with OVA. Pooled unstimulated responses for each group are shown on the left, and the OVA-specific CD4^+^ T cell response for each mouse is shown on the right in both panels. NP-OVA + CpG-vaccinated mice had a robust proportion of CD4^+^ and CD8^+^ T cells producing IFN-γ and TNF-α. Mice vaccinated with OVA-conjugated filariae had OVA-specific CD4^+^ T cells producing IFN-γ and TNF-α but had undetectable levels of cytokine-secreting CD8^+^ T cells. The calculated effect size of OVA-worm (and OVA-NP-OVA + CpG adjuvancy were similar for worms and NP-OVA + CpG at 1.33 in the “CD4^+^ IFN-γ” group and 1.34 in the “CD4^+^ TNF-α” group compared to, 0.99 and 1.2 in the “CD4^+^ IFN-γ” and “CD4^+^ TNF-α” groups, respectively. (**B)** Restimulation of splenocytes from mice immunized with NP-OVA + CpG with biotinylated OVA or native OVA. In contrast to native OVA protein, OVA biotinylated with NHS ester on its lysine residues did not activate CD8^+^ T cells isolated from NP-OVA + CpG-vaccinated mice. (**C**) Restimulation of splenocytes from the filaria-OVA group with filarial lysate. Stimulation with filarial proteins induced the secretion of TNFα by CD4^+^ T cells and IFN-γ by CD8^+^ T cells to the same extent as stimulation with biotinylated OVA. For comparison with nematode (“worm”) lysate, the same worm-OVA values from A (green triangles) are plotted in C.

OVA-conjugated filariae were then inoculated into the mouse dermis to assess the immunization potential of the engineered filaria larvae. As a benchmark, we used an established particulate antigen carrier coupled to CpG, a potent vaccine adjuvant that agonizes TLR9^4,40^. In mice, CpG activates antigen-presenting cells, such as dendritic cells and B cells, and induces the secretion of proinflammatory cytokines, principally interleukin (IL)-12p70 and IL-6, which in turn induce potent Th1 and cytotoxic T lymphocyte responses. CpG is currently being evaluated in clinical trials as an adjuvant for vaccines against cancer and infectious diseases^41^. As expected, vaccination with OVA-conjugated nanoparticles plus CpG (NP-OVA + CpG) resulted in robust OVA-specific CD4^+^ and CD8^+^ T cell responses, as measured by the proportion of splenic T cells producing IFN-γ and tumor necrosis factor TNF-α in response to OVA restimulation (the strong effect size naive of the 1.33 in CD4^+^ IFN-γ^+^ and 1.34 in CD4^+^ TNF-α^+^ groups compared to the naive mice, Fig. 3A). Importantly, vaccination with OVA-conjugated filariae resulted in strong stimulation of worm adjuvancy on OVA-specific CD4^+^ T cell response (effect size 0.99 in the CD4^+^ IFN-γ^+^ group and 1.2 in the CD4^+^TNF-α^+^ group). This observation is particularly notable because these mice did not receive any additional adjuvant, which suggests that filariae act as an adjuvant. Although fewer CD4^+^ T cells responded to OVA delivered by filariae than by nanoparticle vaccine, it should be noted that the OVA dose associated with filarial nematodes was one-fifth that of the nanoparticle vaccine (4 µg vs. 20 µg, respectively). Not surprisingly, we did not observe CD8^+^ T cell responses in OVA-restimulated T cells from the OVA-filaria group, likely because the lysine (K)of the SIINFEKL, the immunodominant MHC class I binding peptide, was masked during the amine-reactive biotinylation (Fig. 3B). CD8^+^ T cell responses were not inhibited in OVA-filaria-vaccinated mice as they responded to the soluble components from naive filariae (Fig. 3C). In contrast, lysine of SIINFEKL is spared during (bio-incompatible) OVA conjugation to -thiol-OVA, which stimulated both CD4+ and CD8 +T cells. CD8^+^ T cells from OVA-filaria immunized C57BL/6 mice retained normal responses as they could be restimulated with the nematode antigens used for the immunization of mice. The absence of a CD8^+^ T cell immune response to the OVA antigen in the filaria-OVA-vaccinated indicated specificity of the OVA-biotin-filariae group served as an internal control for the effect of nematodes on OVA-specific immunity and showed that there was no bystander or nonspecific sensitization of T cells to OVA by chemically modified filarial nematodes.

To address the possibility that vaccination with OVA alone would induce responses similar to those observed following vaccination with worm-OVA, we vaccinated mice with OVA (OVA only) and compared their T cell responses to those of naive mice and mice vaccinated with NP-OVA + CpG. Consistent with our previous experiments, NP-OVA + CpG vaccination resulted in robust OVA-specific CD4^+^ and CD8^+^ T cell responses, whereas naive mice were lacked any observable OVA-specific T cell responses (Supplementary Fig. 2). This allowed us to directly compare the results of the current and previous experiments, confirming the need of adjuvcant in OVA Th1 immunity (Supplementary note). As expected, compared to naive mice, OVA-only vaccinated mice showed no OVA-specific T cell responses, confirming the requirement for an adjuvant to establish T cell immunity.

## Discussion

Interest is growing in the use of various helminths, including filariae, to control allergic and autoimmune reactions^32^. This effort is based on observations that individuals with chronic parasitic nematode infections have reduced rates of autoimmune diseases^42^. The first clinical study testing the hypothesis showed that orally administered porcine whipworm (*Trichuris suis*) eggs reduced the severity of symptoms in patients with inflammatory bowel disease by inducing Tregs and modulatory cytokine production^43^. As of 2016, 28 clinical trials were underway to test the efficacy of the oral administration of *T. suis* and the human hookworm (*Necator americanus*) for the treatment of several autoimmune and allergic disorders^44^. Additionally, various Platyhelminthes, as well as filarial nematodes, have been found to prevent the onset or reduce the severity of type 1 diabetes in mice^45,46^. These therapies are concluded with only a speculative understanding of the immunomodulatory mechanism of intestinal parasites; therefore, it is not surprising that they produce mixed results^47^.

Here, we propose an opposite approach; parasitic nematodes are specially modified to elicit an immune response against specific, the non-filarial antigen. We demonstrate that parasitic nematode can be chemically equipped with an antigen of choice, and together with the intrinsic adjuvant properties of filarial larvae, can be used as a complete vaccine carrier that naturally targets the lymphatic system. The adjuvant properties of filarial larvae, which depend on particulate antigen release^48^, CpG or the parallel discharge of intracellular *Wolbachia* bacteria from dying nematode cells^17^, require in-depth analysis.

The lack of previous reports of using filarial larvae to induce a specific immune response can be partially explained by the technical challenges involved in the genetic modification of filaria. The absence of a free-living stage that could be used to amplify the number of transfected nematodes is a significant obstacle for introducing probes or specific genes such as fluorescent proteins or antigens into parasites^49,50^. Furthermore, sustaining a transgenic colony is difficult because parasites have a complex reproductive cycle with intermediate and definitive hosts (hematophagous arthropods and vertebrates, respectively)^6^. To overcome this limitation, we propose an alternative approach that relies on the chemical modification of reactive primary amines present on the epicuticle and cuticle of the infective larvae^51^. This method enables the nonfatal fluorescent labeling of larvae or, with subsequent bioorthogonal crosslinking, the immobilization of an antigen on the surface of filarial L3 larvae.

The direct modification of filariae with small-molecule fluorophores is a single-step process that was found to produce bright, stable fluorescent labeling. Similarly, the labeling of filariae had no effect on the *in vitro* movement and survival of larvae for up to 5 days. Labeled filariae were also able to migrate *in vivo*, invade lymphatic vessels, and settle in the draining lymph node, indicating that labeling did not affect their lymphatic homing phenotype. The absence of direct protein-labeling toxicity is consistent with the findings of previous reports in which the *in vivo* modification of cell surface amines with isothiocyanate or succinimide esters was minimally toxic to endothelial cells^33,52^ or had no adverse effect on dendritic cell migration^53^.

The immobilization of proteins on the surface of filariae is more complicated than fluorophore labeling because it requires two sequential and noninterfering, but kinetically efficient, reactions that are not toxic to the larvae. The prolonged exposure to low pH borate buffer needed to complete the second step of the bioorthogonal reaction between bifunctional linkers pre-introduced via NHS esters into the OVA (the aromatic hydrazine) and filarial proteins (aromatic aldehyde) was immediately toxic to larvae. Consequently, we used a sandwich technique, in which primary amines of both filariae and OVA were biotinylated with a succinimide ester biotin compound and crosslinked to fluorescently labeled streptavidin (i.e., a bioorthogonal reaction between protein-bound biotin and streptavidin, commercially labeled with a fluorochrome). Similar to the direct fluorescent labeling of filariae, the biotinylation and subsequent crosslinking of streptavidin-OVA protein complexes had no immediate toxic effect on filarial behavior. The tetrameric streptavidin additionally multiplied the number of biotin-binding sites that were saturated in the third incubation step, the second step involving biotinylated OVA. We preferred the amine-conjugation chemistry for biotin crosslinking to OVA because, with 20 primary ε-amino lysine groups on a single OVA protein^54^, this approach maximizes the conjugation of multiple biotins to a single OVA protein. Consequently, the maximized conjugation facilitates the binding of multiple, potentially nonsaturated streptavidin molecules to OVA and the subsequent sandwich amplification of biotin-OVA deposits during the attachment of proteins to the larvae. In addition, amine acylation is a rapid, high-yield reaction^55^, which minimizes the time filariae spend outside their optimal culture medium environment. In contrast, thiol conjugation chemistry was not feasible in this approach because only 4 free cysteine amino acids are present in one OVA molecule^54^. Additionally, cysteines are buried within the protein, and additional denaturation and renaturation (solubilization) steps are required^55^. However, the modification of overrepresented amino acids, such as lysine, increases the risk of destroying the epitope that is recognized by MHC molecules. Indeed, CD8^+^ T cell activation by OVA protein depends on the lysine in the OVA immunodominant SIINFEKL epitope being unmodified when presented in the context of MHC I molecules^56^. As expected, the OVA-induced CD8^+^ T cell response was lost after the biotinylation of OVA. Nevertheless, the CD4-dependent Th1-type immune responses to OVA were not affected by biotinylation because the ISQAVHAAHAEINEAGR peptide, the dominant OVA epitope, presented in the context of MHC II molecules^57^, is not modified by amine crosslinking chemistry. As anticipated, CD4^+^ but not CD8^+^ T lymphocytes from mice immunized with OVA-filaria could mount a Th1 immune response as determined by the production of IFN-γ or TNF-α in the biotinylated OVA-restimulated CD4^+^ T lymphocytes.

To date, the early stages of infection have not been imaged because filarial labeling is a technically challenging process^49^. Therefore, the presented method of intravital fluorescent labeling of infective larvae may become an indispensable tool for imaging the postinfection fate of filarial and possibly other parasites. Moreover, the biotinylation of nematode proteins and subsequent labeling of live larvae with streptavidin opens imaging opportunities beyond the limits of fluorescence microscopy. As virtually any tracer can be attached to streptavidin without affecting its specificity^58^, this labeling method should be readily adapted for high resolution (˂1 mm) whole-body imaging techniques, such as magnetic resonance imaging (gadolinium-labeled streptavidin), ultrasound imaging (streptavidin-functionalized microbubbles), or positron emission tomography (^124^I and ^64^Cu-labeled streptavidin)^59^ allowing the visualization of, for example, the infection routes of intestinal parasites.

These experiments signal the potential of an alternative method for inducing an immune response against customized, non-nematode antigens. Immunization with OVA protein without an adjuvant does not provoke any immune response^2,4^. In contrast, the filaria-delivered antigen-induced an immune reaction and, unlike the benchmark nanoparticle-CpG vaccine, required no additional immunostimulatory adjuvant. The immune response induced by a dose of OVA on filaria that was one-fifth that of the nanoparticle vaccine demonstrated an evident dose-sparing effect of the worm-OVA vaccine. Importantly, *Wolbachia*, bacteria from the Rickettsia family, combine the advantages of a robust live Th1 stimulant with the absence of direct pathogenicity to humans; they are obligate intracellular symbionts of invertebrates, and they cannot proliferate and survive in mammals^60^.

The use of filaria as an antigen and adjuvant carrier highlights a unique feature, specifically, antigen exposure in the context of a natural inflammatory environment. Hence, T cell stimulation by nonspecific filarial antigens may be advantageous when, for example, a response to a target antigen can benefit from complex local inflammation^61^ or the stimulation of innate immunity^62^. We have shown that with simple manipulations, filarial parasites can be recruited as natural vaccine carriers, integrating the targeting of the lymphatic system and adjuvant formulation. Possible replacement of filaria larvae with nonpathogenic, free-living nematodes should increase the safety and, therefore, the applicability of this technique^22^.

The method of chemical immobilization of an antigen on filaria served the purpose of verifying the concept. However, due to its effects on the structure of protein epitopes, it should be substituted with more neutral approaches, such as transgene expression in nematode cells. Additionally, the focus on Th1-type CD4^+^ T cell responses limits the conclusions that can be drawn from our study. For example, although Th1-type cellular immune responses have previously been observed in filariasis patients with filarial lymphedema^63^, infection with helminths is typically associated with polarization towards a classical Th2-type humoral response or, in some cases, a more refined CD4^+^ phenotype, such as Th9, Th17, Th22 or immunosuppressive Tregs (reviewed in^64^). However, the long-term presence of adult filariae and their microfilariae offspring in patients with filariasis would be expected to exert a different effect on the immune system than the model proposed here, where brief exposure to dying infective filaria larvae and their intracellular adjuvant is used to stimulate the immune system. Further experiments are needed to define the mechanism by which filarial larvae stimulate immunity against nonfilarial antigens and the resulting CD4^+^ T helper cell phenotype. However, we show here that harnessing the intrinsic adjuvancy of a nematode carrier offers an alternative method of immunization that might lead to the development of vaccines producing pronounced cellular immunity.

## Materials and Methods

### Ethics statement

This study was carried out in strict accordance with the Swiss Animal Protection Act, the ordinance on animal protection, the ordinance on animal experimentation, and the Veterinary Authority of the Institutional Animal Care and Use Committee of the University of Chicago. The protocols were approved by Commission de surveillance de l’Etat de Vaud. Animal immunization experiments were carried out in accordance with EU Directive 2010/63/UE and the French legislation “Décret no 2013-118, 1^er^ février 2013, Ministère de l’Agriculture, de l’Agroalimentaire et de la Forêt.” The animal experiments were approved by the Ethics Committee of the Museum National d’Histoire Naturelle (MNHN) and by the “Direction départementale de la cohésion sociale et de la protection des populations.”

### Reagents

Endotoxin-free ovalbumin (OVA) and CpG-B 1826 were purchased from InvivoGen (San Diego, CA); fetal bovine serum (FBS), Dulbecco’s modified Eagle’s medium (DMEM), HEPES, penicillin/streptomycin/amphotericin B solution (100×), FITC, 5 TRITC, and EZ-Link sulfo-NHS-biotin, Zeba Spin columns, DMSO (dimethylsulfoxide) were purchased from Thermo Fisher Scientific (Waltham, MA); the biotinylated anti-collagen IV antibody and Streptavidin-Alexa Fluor 488 and -Alexa Fluor 647, Alexa Fluor 647-NPs, Alexa Fluor 647 C2 Maleimide were from Invitrogen (Carlsbad, CA); Hartmann’s solution was from Braun (Melsungen, Germany); maleimide-DY-647 was from Dyomics (Jena, Germany); sulfo-S-HyNic and sulfo-S-4FB were from TriLink Biotechnologies (San Diego, CA); CpG-B 1826 from InvivoGen (San Diego, CA); Sephadex G-25 columns were from GE Healthcare (Glattbrugg, Switzerland). Other common reagents were from Sigma-Aldrich (St Louis, MO).

### Maintenance of L3 infective filariae

The *L. sigmodontis* life cycle was maintained as previously described^65^. Briefly, a patent filarial infection was established in gerbils (*Meriones unguiculatus*) at the MNHN laboratory in Paris, France. Infective third-stage larvae (L3) were isolated by dissection of the mite vector *Ornithonyssus bacoti*, counted, and placed in cell culture medium (H-DMEM; 4.5 g glucose/L, DMEM with 25 mM HEPES, 400 U/ml penicillin, 400 U/ml streptomycin, 1 μg/ml amphotericin B, pH 7.5) before labeling under 5% CO_2_ at 37 °C.

### Isolation of free-living (soil-derived) nematodes

Free-living nematodes, including plant-parasitic roundworms, were isolated from soil samples using the Baermann funnel method^66^. Soil samples were collected during the summer season from continuously monitored grassland that is not visited by livestock, pets, or wild animals. To remove large, insoluble particles, 100 g aliquots of the soil samples were mixed with 300 ml of deionized water and passed through a funnel-shaped No. 20 sieve (850 μm mesh size). The same mesh sieve was placed within a larger funnel fitted with 5 cm of rubber tubing, which was sealed with a hemostat. A Kleenex tissue was placed on the screen, and deionized water was poured into the funnel until it covered approximately half of the fitted screen. Subsequently, soil samples were poured onto the screen, and nematodes were collected in the first 50-ml fraction from the rubber tubing 48 hours later. Nematodes were spun down at 500 × g and hand-picked from the remaining 5 ml of water with a 200 μl pipette. Collected nematodes were kept at 4 °C, and prior to staining with TRITC, the nematodes were transferred to phosphate-buffered saline (PBS; pH 7.4).

### Labeling of filarial L3 larvae and free-living nematodes with FITC, TRITC or EZ-Link sulfo-NHS-biotin

Stage 3 (L3) *L. sigmodontis* larvae were washed in 3 ml of Hartmann’s solution. Prior to labeling, 25 mg/ml of FITC or TRITC stock solution in DMSO, cell culture-grade) was diluted to 250 μg/ml in Hartmann’s solution and passed through a 0.45-μm filter. The EZ-Link sulfo-NHS-biotin stock solution was made by dissolving the EZ-Link sulfo-NHS-biotin directly in Hartmann’s solution at a concentration of 250 μg/ml. Next, 0.5 ml of FITC-, TRITC-medium, or EZ-Link sulfo-NHS-biotin was immediately mixed with 100 μl of Hartmann’s solution containing 100 to 300 filarial larvae for 15 minutes. Later, larvae were washed three times in a new vial containing 3 ml of Hartmann’s solution and five times in H-DMEM with 20% FBS.

### OVA labeling and bioorthogonal conjugation to filariae

#### Nanoparticle synthesis and conjugation with OVA

Thiol-immobilization of denatured OVA to nanoparticles. Pluronic-stabilized PPS-NPs were synthesized by emulsion polymerization and surface-functionalized, as previously described (7). Alexa 647-NPs were labeled with Alexa Fluor 647 C2 Maleimide, and free dye was removed at the end of the reaction by gel filtration through Sepharose CL6B exclusion matrix. For antigen conjugation, OVA and NPs were incubated for 6 h at room temperature in the presence of guanidinium hydrochloride to expose by partial unfolding the free thiols on the protein.

#### Amine substitution with aromatic hydrazine (S-HyNic) and an aromatic aldehyde (S-4FB)

For OVA thiol labeling, 10 mg of protein was mixed with 6 M guanidine hydrochloride and 4 equivalents of DY-647-Maleimide that reacts with exposed free thiols. The fluorescently labeled OVA was dialyzed from guanidine HCl and unconjugated dye by size exclusion through a Sephadex G-25 column. For conjugation to filaria, water-soluble sulfo-S-HyNic and sulfo-S-4FB crosslinkers were used. All reactions were performed at room temperature. Briefly, 5 mg of previously fluorescently labeled OVA was mixed with a 10-fold molar excess of sulfo-S-HyNic in 100 mM phosphate buffer, 150 mM NaCl at pH 8.0. The protein was purified through a Zeba Spin column equilibrated in 100 mM phosphate buffer, 150 mM NaCl at pH 6.0, to remove any excess unbound crosslinker. In parallel, 30 filariae were labeled with 5 mg of sulfo-S-4FB in PBS, and the free crosslinker was removed with 5 repeated H-DMEM washes. Once the OVA and filariae were functionalized, both components were mixed and left to react for 1 hour in 150 mM NaCl at pH 6.0 buffer. Filariae were then washed 5 times with 3 ml of H-DMEM supplemented with 20% FBS to remove free OVA protein followed by 2 washes with Hartmann’s solution and then imaged on a Leica fluorescence stereomicroscope.

#### Streptavidin sandwich method

All reactions were performed at room temperature. For OVA amine modification with water-soluble EZ-Link sulfo-NHS-biotin, 10 mg of OVA protein was mixed with 10 molar equivalents of biotinylation reagent in PBS (pH 7.5), and the reaction was continued for 2 hours at room temperature. Unconjugated biotin was removed from the solution by size exclusion through a Sephadex G-25 column. The same reagent was used to label filariae with biotin. Larvae were first washed with 3 changes of PBS and reacted for 15 minutes with 250 μg/ml freshly prepared EZ-Link sulfo-NHS-biotin in PBS. After 3 subsequent washes in Hartmann’s and 5 washes in H-DMEM supplemented with 20% serum, the larvae were incubated with 400 μg of Alexa 488-streptavidin for 10 minutes. After being washed 5 times with H-DMEM with 20% serum, larvae were washed twice with Hartmann’s solution and imaged on a Leica fluorescence stereomicroscope. Larvae were then reacted for 10 minutes with biotinylated OVA and washed 5 times with H-DMEM with 20% serum. After the fifth wash, the larvae were incubated with 400 μg/ml of Alexa 647-streptavidin in H-DMEM for 10 minutes. After being washed 5 times with H-DMEM with 20% serum, the larvae were washed twice with Hartmann’s solution and again imaged on a Leica fluorescence stereomicroscope in green (488 nm) and far-red (647 nm) channels to determine the amplification of fluorescent labeling of the filariae. The larvae were then incubated for a second time with 2 mg/ml biotinylated OVA, washed 5 times with H-DMEM with 20% FBS, twice with Hartmann’s and injected into mice.

### Densitometric determination of the filaria-bound OVA with Western blot

After being sandwich labeled with OVA-streptavidin, 60 filaria larvae were transferred to pure water and frozen at −20 °C. Thawed filariae and serial dilutions of biotinylated OVA and pure OVA were lysed with the addition of 5 × SDS-PAGE sample buffer (350 mM Tris, pH 6.8, 10% SDS, 0.5 M DDT, 50% glycerol, 10 mg/ml bromphenol blue) supplemented with 10% Triton-X-100 and 25 mM EDTA. Each filarial sample was sonicated until it boiled, cooled to room temperature, and centrifuged at maximum speed for 5 minutes. The soluble fraction of nematode proteins was collected, and together with two biotinylated OVA serial dilution standards, they were heated for 7 minutes at 95 °C. After cooling to room temperature, samples were supplemented with 200 mM iodoacetamide and loaded into wells of the same stacking gel. Samples were resolved on 12% acrylamide gel and transferred with semi-dry blotting to a 0.2 µm 100% nitrocellulose membrane (Biorad). The membrane was blocked at 42 °C for 1 hour with 5% bovine serum albumin in Tris-buffered saline (TBS) containing 0.1% Tween- 20. The membrane was incubated with 10 mg/ml HRP-conjugated streptavidin (ThermoFisher), and the OVA bands were visualized on photographic film with an ECL reagent kit (Pierce). Densitometric measurements were performed on nonsaturated bands, and the filaria-derived OVA concentration was calculated from a serial dilution of biotinylated OVA reference bands.

### Larval inoculation and whole-mount tissue imaging

Approximately 30 fluorescently labeled larvae in 30 μl of Hartmann’s solution were loaded into U-100 insulin syringes from the plunger side and allowed to settle at the needle outlet for 10 minutes. Twenty microliters of the larval suspension were then injected intradermally into the top dorsal region of each mouse’s ear. In total, 14 mice were used in these experiments.

Whole-mount staining and imaging were performed essentially as described before^34,67^. Mice were fixed by intracardiac perfusion. Blood was first removed at a constant pressure of 100 mm Hg with 20 ml of Ringer-lactate solution (Hartmann’s solution) supplemented with 25 mM HEPES and 0.1% glucose. The mice were then perfused with 25 ml of zinc fixer (39 mM Zn(CF_3_COO)_2_, 37 mM ZnCl_2_, 4.5 mM CaCl_2_, 60 mM glycine, pH 6^67^). Ears and lymph nodes were dissected and placed in ice-cold zinc fixer for at least 24 hours. Later, ventral skin, muscle, and cartilage were removed from the ear, and dorsal skin and lymph nodes were postfixed for another 24 hours in zinc fixer with 0.1% Triton-100. Tissues were then washed twice in TBS (25 mM Tris, 140 mM NaCl, pH 7.5), briefly incubated in 0.5% casein in TBS, and labeled for 24 hours with 10 μg/ml biotinylated primary anti-collagen IV antibody. After being washed in TBS-0.1% Tween® 20, the tissues were incubated with Alexa 488- or Alexa 647-streptavidin for 24 hours. Washed tissues were clarified in Murray’s clear (1:2 benzyl alcohol/benzyl benzoate^68^), mounted on a glass slide, and imaged using a Leica SP5 confocal microscope equipped with a white-spectrum laser or an Olympus IX82 microscope equipped with an Olympus disk spinning unit, Spectra X light engine from Lumencor and Orca-Flash 4.0 v2 from Hamamatsu. Optical sections, maximum intensity projections (MIPs), or overlay surface masks were generated from image stacks using Imaris 7.1 (Bitplane AG, Zürich, Switzerland).

### Live imaging of larvae in the skin

The staining of ear intravital skin was performed as previously described^34^. Mice were anesthetized with 3% induction and 1.5% maintenance concentrations of isoflurane. Ventral skin, muscle, and cartilage of the ear were partially removed, leaving only the top fragment of the ventral skin attached to the dorsal skin flap. The dorsal skin was then stained for 30 minutes with 10 μg/ml biotinylated anti-collagen IV antibody or anti-Lyve1 antibody, and an anti-podoplanin antibody in Hartmann’s solution supplemented with 2 mg/ml aprotinin. After the tissue was washed 5 times with Hartmann’s solution, the primary antibodies were detected with 10 μg/ml fluorescent streptavidin or the appropriate fluorescent secondary antibodies. Thirty larvae were then either applied to the prelabeled exposed ear dermis or injected intradermally in the top dorsal skin located above the collagen IV-stained dorsal skin. Immediately after inoculation, filaria larvae and stained tissue were imaged every 30 seconds using a Leica M205 FA fluorescent stereomicroscope.

### Vaccinations

C57BL/6 mice were injected subcutaneously in a footpad with 35 filariae conjugated to OVA (4 µg dose, 6 mice). As a positive control, another group of mice received an intradermal injection of 20 µg of OVA conjugated to synthetic nanoparticles with 2.5 µg of CpG-B 1826 (5 mice) as an adjuvant according to previously published protocols^4^, and 4 mice served as negative controls were injected with PBS or 20 µg of OVA. Mice received a boost of the same vaccine formulations 1, 2, and 3 months later. One month after the last boost, mice were euthanized, and spleens were collected and passed through a 60-µm cell strainer for further T cell restimulation.

### Antigen-specific restimulation

Splenocytes were seeded at 3 × 10^6^ cells/well in round-bottom 96-well tissue culture plates in Iscove’s Modified Dulbecco’s medium supplemented with 10% FBS and 1% penicillin/streptomycin. Cells were restimulated with 100 μg/ml OVA grade VI (CpG group), or where indicated, with biotinylated OVA (NP-OVA CpG vaccination group) or filariae lysate (30 filariae frozen and sonicated on ice in 100 μl of cell culture medium). Pooled samples from mice in each group were left unstimulated to measure background levels of cytokines. After 3 hours, the cells were treated with brefeldin A (5 μg/ml) to block cytokine secretion and then incubated for another 3 hours. The cells were stained according to standard intracellular cytokine staining protocols and were analyzed for cytokine production by flow cytometry^4^.

### Statistical analysis

Statistical comparisons of 2 treatment groups relative to the control (naive mice) group were made using one-way ANOVA followed by Dunnett’s post hoc test (Fig. 3 and Supplementary Fig. 2). An unpaired *t*-test with Welsh’s correction for unequal variance was used when comparing two groups. Differences, where p-values were less than 5%, are joined by a vertical line. Tests were computed as two-tailed assumptions. The effect sizes were calculated for the comparisons between NP-OVA + CpG and worms relative to the naive control mice (Fig. 3A). The population standard deviation was estimated from the pooled standard deviations of the compared groups using (1) Cohen’s index: SD_pooled_ = SD $\,=\sqrt{\frac{(S{D}_{1}^{2}+S{D}_{2}^{2})}{2}}$. Statistical analyses were performed using GraphPad Prism (ver. 7.1 for Windows, GraphPad Software, La Jolla California USA, www.graphpad.com).

## Supplementary information

Video 1.

Video 2.

Video 3.

Video 4.

Video 5.

Video 6.

Video 7.

Supplementary info, figures and video description.

## Data Availability

All data generated or analyzed during this study are included in this published article (and its Supplementary Information files). Source datasets generated and/or analyzed during the current study are available from Figshare online repository under 10.6084/m9.figshare.11407239.
