# Supplementary material for: Adjuvant-free immunization with infective filarial larvae as lymphatic homing antigen carriers
Source: Sci Rep. 2020 Jan 23;10:1055. doi: 10.1038/s41598-020-57995-8 (PMC6978462; doi:10.1038/s41598-020-57995-8)
Supplement: Supplementary file 8 — Supplementary info, figures and video description. [file 41598_2020_57995_MOESM8_ESM.pdf]

Supplementary Information

Adjuvant-free immunization with infective filarial larvae as lymphatic homing antigen carriers

Catherine Card[1] David S Wilson[2], Sachiko Hirosue[1], Marcela Rincon-Restrepo[1], Alexandre de Titta[1], Esra Güç[1], Coralie Martin[3], Odile Bain[3†] & Melody A Swartz[1,2] Witold W Kilarski[1,2,✉]

1 Institute of Bioengineering and Swiss Institute for Experimental Cancer Research (ISREC), École Polytechnique Fédérale de Lausanne (EPFL), Lausanne, Switzerland

2 Institute for Molecular Engineering, University of Chicago, Chicago, IL, USA

3 UMR7245, MCAM, Museum National d'Histoire Naturelle, Paris, France

† Deceased

✉ - Corresponding author: wkilarski@uchicago.edu

Supplementary figures

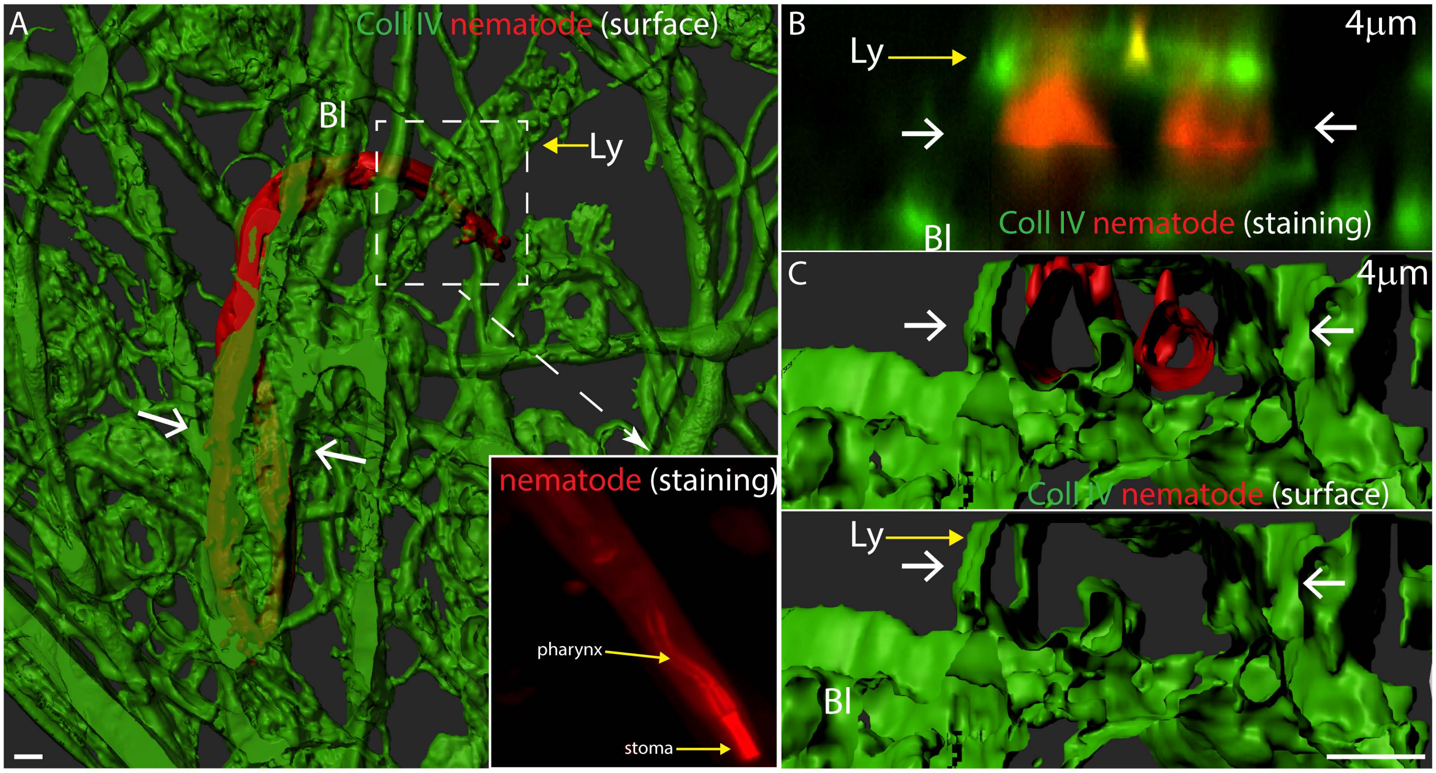

Supplementary Figure 1. TRITC-labeled saprophytic nematode entered lymphatic pre-collector

Surface mask from the z-stack of the confocal images of collagen IV (green) and free-living, soil-derived nematode (red) that was injected in the dorsal dermis of the mouse ear. **A.** Free-living nematode entered the lymphatic vessels (Ly). Lymphatic identified by its morphology is partially masked by a large blood vessel. **Inset**. Head of the nematode showing characteristic for bacterivore nematode distinction between simple, unmodified stoma and pharynx. Arrows point to the location of the optical cross-section shown in B-C. **B-C.** Optical 4μm cross-section of the image stack from A showing intra-lymphatic localization of the free-living bacterivore. B. Fluorescent intensities. C. Surface masks (unified intensities). C. Bottom. The same mask as in C. Top, without the nematode channel. Scale bars, 20 μm.

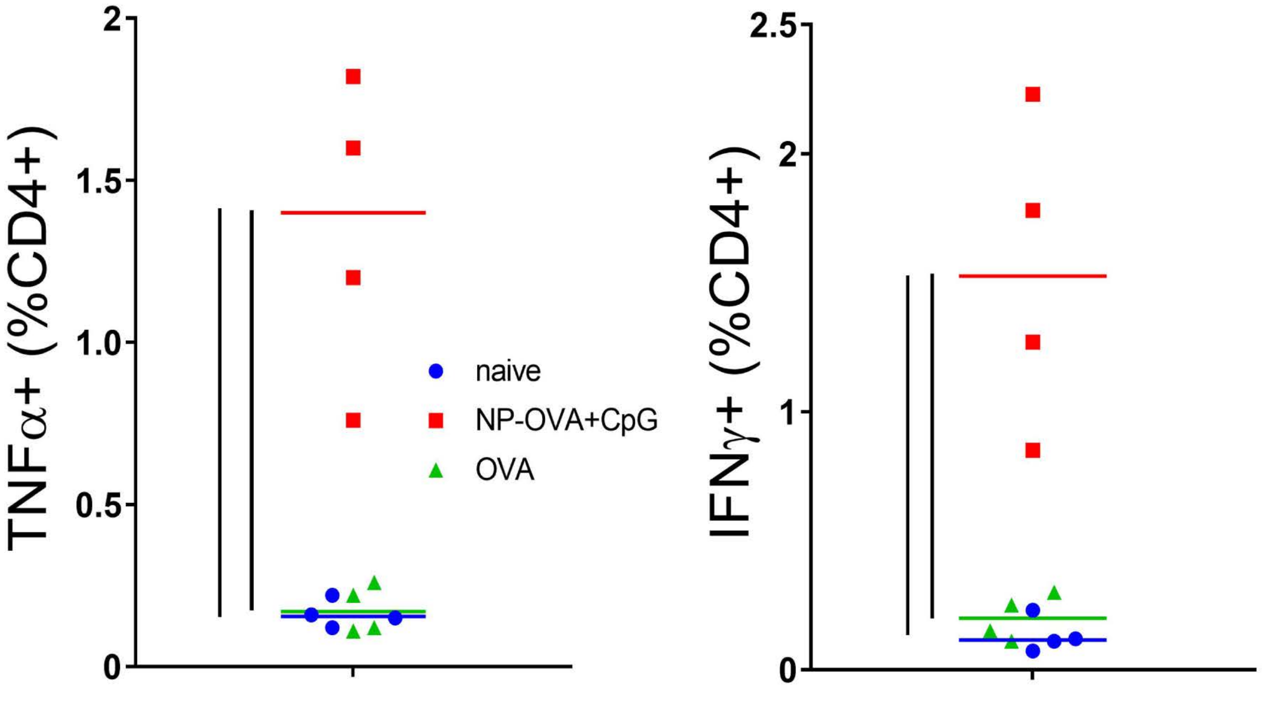

Supplementary Figure 2. Vaccination only with ovalbumin does not stimulate OVA-specific T-cell responses

Vaccination with OVA protein only was performed according to the schedule of the experiments presented in Figure 3. To match the new OVA-only control group with the initial results, two control groups – naive (negative control) and OVA-conjugated nanoparticles + CpG mice (NP-OVA+CpG, positive control) – were repeated together with the OVA-only group. Splenocytes were isolated from naive (PBS) mice or mice vaccinated with either NP-OVA+CpG or only OVA protein and restimulated with OVA for 6 hours. OVA-restimulated $CD4^+$ T cells from mice immunized with NP-OVA+CpG showed a robust response in the production of TNFα and IFNγ ($p<0.001$, ANOVA), whereas the response of $CD4^+$ T cells from mice vaccinated with OVA-only, was indistinguishable from the naive group ($p=0.68$ (IFNγ) and $p=0.99$ (TNFα)).

Videos

Videos are encoded with free Xvid MPEG-4 Video Codec (xvidcore.dll version 1.3.4) from www.xvid.com) encoder and contained in the AVI format).

Video 1. FITC-labeled filariae are swarming in cell culture media. Snapshots were taken every 0.2 s; 2 seconds video played at 10 fps.

Video 2. Phototoxicity of FITC-labeling. FITC-labeled filariae exposed to constant blue (fluorescein excitation) light died after 15 s of imaging. Snapshots were taken every 0.4 s; 6 seconds video played at 10 fps.

Video 3. Burrowing phenotype of labeled larvae entering the dermis. TRITC-labeled larvae overlaid on tissue stained for lymphatic markers, Lyve1, and podoplanin (green). Note, as Fc-receptors were not pre-blocked, this staining also marked tissue macrophages that bind fluorescent immune complexes. Live dermis surgically exposed before the staining. Snapshots were taken every 2 min; 3 seconds video played at 6 fps.

Video 4. Burrowing phenotype of labeled larvae in the dermis. FITC-labeled filariae injected in the ear dermis. The imaging was done through the intact skin. Snapshots were taken every 0.2 s; 4 seconds video played at 10 fps.

Video 5. Extended imaging of TRITC-labeled filariae that stuck within the dermis. In contrast to fluorescein (Video 2), imaging associated toxicity from rhodamine labeling was less apparent to larvae. Vessels and nerves of the live tissue stained green for collagen. 7.5-hour video. 17 seconds video played at 50 fps.

Video 6. Non-uniform labeling (bright patches) of filariae after S-4FB and S-HyNic-OVA-647 conjugation. Dying or dead larvae are weakly, or non-motile. Snapshots were taken every 0.5 s; 2 seconds video played at 5 fps.

Video 7. Alexa-647 stained OVA-Alexa-488 labeled, biotinylated larvae were alive 3 days after labeling. Filaria larvae imaged in 647 channel 1 day after in vivo biotinylation, staining with Alexa-488 streptavidin, subsequent labeling with biotinylated OVA, and staining with Alexa-647 streptavidin-biotinylated OVA complexes. In contrast to small hydrophobic fluorophore staining (FITC or TRITC), streptavidin-OVA labels larval cuticle but not intestine or internal structures. Some non-motile dying larvae can be already seen. High-intensity spots are results of multiplying the fluorescent signal from overlayed larvae. Snapshots were taken every 0.2 s; 5 seconds video played at 10 fps.

Microscopy equipment and settings

Intravital epifluorescence imaging

Intravital imaging was done with M250 FA (Leica Microsystems CMS GmbH, Wetzlar, Germany) motorized fluorescence stereomicroscope, equipped with 1× lens (linear system magnification from 7.5× to 160×) or 2× lens (linear system magnification from 15.6× to 320×) and DFC 350 FX camera controlled by LAS AF 2.9 software (Leica Microsystems). The exposition of 12-bit images was adjusted to fill the dynamic range of an image. For fluorescence imaging, the range of camera grey-scale values was set from 0% (minimum) to 5.1% (maximum) and set gamma correction to 1. With gain set to minimum (1) and maximum power of the Leica fluorescence light source (EL6000), the exposure time was adjusted to fill the 12-bit dynamic range without clipping highlights, midtones, and shadows, and was between 0.2 and 2 seconds. Camera frame rate was 20 MHz with image resolution 1000x1200 pixels. Original Leica lif files are available upon request.

Optical sectioning of thick whole-mount preparation of cleared tissue

Leica SP5 confocal microscope

Confocal images in Figures 1-3 and Supplementary Figures 2-4 were taken with Leica SP5 inverted confocal microscope equipped with beam-splitting prisms and the White Laser light source tunable from 470nm to 670nm in 1nm increments. 12-bit images were collected at 1012 x 1024 resolution with HC PL APO correction ring 20x/0.75 immersion lens or HC PL APO 63x/1.40 oil objectives. At optimal (following the Nyquist theorem) Z step (0.8 µm for the 20x lens and 0.25 µm for the 63x lens), XY planes were scanned with the resonance scanner speed of 8000 HZ, each field was averaged 48 times, and the signal was collected simultaneously by two HyD detectors. Images were processed with Imaris 7.4 software (Bitplane) and stored as Imaris (.ims) files (available upon request).

Supplementary Note

Accounting for different number of animal in naive and OVA-only control groups

Because a statistical model cannot conclude the lack of difference between groups, for the comparison of naive and OVA-only controls, we turned to statistical power calculations for naive and OVA-only control groups. Since we had fewer mice in our current experiment (n=4/group) than our previous experiment (n=6/group), the argument could be made that the experiment was not sufficiently powered to observe a difference between naive and OVA-vaccinated mice. To account for that, we therefore performed a statistical power calculation to determine whether we would be sufficiently powered to observe a difference between naive and OVA-vaccinated groups at n=6/group and standard deviations of the observed CD4+ T cell responses (Control group I: 'naive': $\mu_{TNF\alpha} = 0.16\%$, $\mu_{IFN\gamma} = 0.13\%$, $\sigma_{TNF\alpha} = 0.04$, $\sigma_{IFN\gamma} = 0.068$; Control group II 'OVA-only': $\mu_{TNF\alpha} = 0.13\%$, $\mu_{IFN\gamma} = 0.19\%$, $\sigma_{TNF\alpha} = 0.07$, $\sigma_{IFN\gamma} = 0.08$), we would have, at least, a 14% chance of detecting a difference in TNFα and a 26% chance of detecting a difference in IFNγ responses between naive and OVA-vaccinated mice at n=6/group. We observed much stronger differences in $CD4^+$ T cell responses between naive and worm-OVA-vaccinated mice at n=6/group (Fig. 3 and Supplementary Fig. 1), which provides further evidence of an adjuvant effect of the filariae.
